# Blue’s Elegy

**Published:** 1885-12

**Authors:** Hippocrates Blue

**Affiliations:** Austin, Tex.


### BLUE'S ELEGY.

### THE BILIOUS DOCTOR TO HIS LADY-LOVE.

BY F. E. D., AUSTIN, TEXAS.

(Manufactured on our own Patent, Automatic, Verse-Making Machine.)

Your life leads down by peaceful, tranquil rivers,
Whose shady bank the cool sea breeze invites ;
While mine, alas, is spent midst torpid livers,—
And all such bilious, melancholy sights.

To you, the perfumed air is rich with sounds,
As sweet as when first Sappho's harp was strung;
While I, in sun and dust must take my weary rounds
To feel a pulse, or view a coated tongue.

The choicest books beguile your leisure hours,
And soothe you, like the "music of the spheres;"
But woe, is me ! I spend my feeble powers
'Midst fever's fervid heat, or checking diarrhœas.

You sleep in peace on soft and downy beds,
And dream, perhaps, of flowers in sunlit lands;
While I, no doubt, am soothing aching heads,
Or nobly giving aid by pulling hands !

Your lovers kneel before you in rapturous adoration,
And tales of love in mellifluous measures pour;
Creditors besiege me; they are my abomination;
And moneyless patients daily throng my office door.

Thy gentle pen, anon, the choicest thought indicts,
That most fond memory e'er kindly fosters;
Prescriptions I, with stubby pen must write,
*Recipe; misce et fiat haustus.*

Treasure I bring thee not, to pride's exactions fill, [tion;
Nor offer thee, as I could wish, a handsome marriage por-
Wilt thou despise my only store—a pill ?
Or deign to take perchance, a pharmaceutical lotion ?

Alas, alas, my lady-love, I tire indeed of these
  Old scaly scalps of seborrhœa and eczematous hands;
Let's trim our sails to catch an outward breeze,
  And *endosmose* in pleasant foreign lands,—

Away beyond the seas, on some peaceful star-lit isle,
  Where rythmic wavelets break on coral strands,
There'll be no more of fever, pus, nor bile,
  As down the happy years we'll pull *each others* hands.

*Austin, Tex., Dec.* 10, 1885. HIPPOCRATES BLUE, M. D.

